# Population awareness of risks related to medicinal product use in Vientiane Capital, Lao PDR: a cross-sectional study for public health improvement in low and middle income countries

**DOI:** 10.1186/s12889-015-1948-2

**Published:** 2015-06-27

**Authors:** Céline Caillet, Chanvilay Sichanh, Lamphone Syhakhang, Cyrille Delpierre, Chanthanom Manithip, Mayfong Mayxay, Maryse Lapeyre-Mestre, Paul N Newton, Anne Roussin

**Affiliations:** Faculté de Médecine, Equipe de Pharmacoépidémiologie UMR 1027 INSERM-Université de Toulouse III, Service de Pharmacologie Médicale et Clinique, Centre Hospitalier Universitaire de Toulouse, 37 allées Jules Guesde, 31000 Toulouse, France; WorldWide Antimalarial Resistance Network, University of Oxford, Wellington Square, OX1 2JD Oxford, UK; Food and Drug Department, Ministry of Health, Simuang Road, Vientiane Capital, Lao People’s Democratic Republic; Faculté de Médecine, Cancer et maladies chroniques, UMR1027 INSERM- Université de Toulouse III, 37 Allées Jules Gusede, 31000 Toulouse, France; Faculty of Pharmacy, University of Health Sciences, P.O.Box 7444, Samsenthai Road, Vientiane, Lao People’s Democratic Republic; Lao-Oxford-Mahosot Hospital-Wellcome Trust Research Unit, Microbiology Laboratory, Mahosot Hospital, FaNgum Rd, Vientiane, Lao People’s Democratic Republic; Faculty of Postgraduate Studies, University of Health Sciences, P.O.Box 7444, Samsenthai Road, Vientiane, Lao People’s Democratic Republic; Centre for Tropical Medicine and Global Health, Nuffield Department of Medicine, Churchill Hospital, University of Oxford, Wellington Square, OX1 2JD Oxford, UK

**Keywords:** Lao PDR, Low- and middle-income countries, Public health, Population, Awareness, Safety, Medicinal products

## Abstract

**Background:**

While essential medicines have been made more available in all but the most remote areas in low and middle income countries (L/MICs) over the past years, inappropriate and incorrect use of good quality medicines remains a key impediment for public health. In addition, as medicines have a potential to cause harm (medicine risks), adequate awareness by medicine users of the risks of adverse reactions is essential, especially as self-medication is common in L/MICs. This study aimed to investigate the awareness of Lao residents regarding medicine risks in Vientiane Capital, Lao People’s Democratic Republic.

**Methods:**

Face-to-face interviews using structured questionnaires of 144 residents older than 16 years were carried out in 12 randomly selected villages out of the 146 villages of Vientiane Capital with at least one health facility.

**Results:**

The respondents were mainly (85.0 %) the heads of households or their husband/spouse . The majority of the respondents were unaware (61.8 %) of medicine risks. Compared to residents living in the urban district of Xaysetha, living in peri-urban and even more in rural areas were identified as factors associated with being unaware of medicine risks [adjusted odds ratio (aOR) =3.3, 95 % Confidence Interval (CI) = 1.1–9.4]) and aOR =7.5 (95 % CI = 2.3–24.2), respectively]. In addition, more than half of the respondents had never heard of poor quality medicines, with a higher rate in rural/peri-urban compared to urban districts (55.6 % vs 38.9 %, respectively, *p* = 0.02). Finally, approximately one third of all respondents thought that traditional medicines could not cause harm.

**Conclusions:**

Overall, these results suggest a lack of awareness about medicinal product risks. Differences according to the place of residence are apparent and could be partly explained by a lower level of training of healthcare providers in contact with the population in the rural districts in particular. Communication on medicinal product risks to patients through well-trained healthcare providers could probably make a valuable contribution towards the appropriate use of medicines in L/MICs.

## Background

Over the past 20 years, the rapid expansion of health markets, especially in low and middle income countries (L/MICs) in Asia and in Africa, has made many medicines available in all but the most remote areas [1]. This growing market, although providing pharmacological treatments to many patients, has raised problems of drug safety associated with inappropriate and incorrect use of medicines. The World Health Organization (WHO) estimates that more than 50 % of all medicines are prescribed, dispensed or sold inappropriately and that half of all patients fail to take medicines correctly [2].

In L/MICs countries, medicine users themselves are key actors for an appropriate and correct use of medicines. Indeed, self-medication is a common practice, partly because most medicines are available without any prescription [3]. Moreover, people commonly use traditional medicines for which safety is relatively unknown [4, 5]. Finally, people in L/MICs countries are more and more exposed to the growing market of poor quality medicines [6]. Consequently, the threat of adverse drug reactions (ADRs) is a major issue in these countries.

In a survey performed in Zimbabwe, 65 % of the interviewees (adults members of households) were unaware of over-the-counter (OTC) medicine risks [7]. Developing people’s awareness of the potential harmful effects of medicinal products may be particularly relevant to improve their appropriate and correct use.

Lao People’s Democratic Republic (Lao PDR) is a lower-middle income country in South-East Asia. Although efforts have been made to improve the use of medicines since the adoption of the National Drug Policy in 1993 [8], some important issues remain, such as the low quality of private pharmacy services [9], common self-medication [10, 11], inappropriate prescribing and dispensing of medicines in public health facilities [12], and the existence of poor quality medicines in the market [13].

The aim of this study was to investigate the awareness of the population on medicine risks in Vientiane Capital, Lao PDR. In addition, we aimed to study the awareness of the population on traditional medicine risks and on the existence of poor quality medicines.

## Methods

### Study population

This cross-sectional study was conducted between August and November 2012 in Vientiane Capital. Vientiane Capital is composed of nine districts and 483 villages and had an estimated population of 797,130 inhabitants in 2012 [14].

With the aim to get a representative study population of Vientiane Capital districts, two urban districts (Xaysetha and Sikhottabong) were randomly selected among the four urban ones, one (Naxaithong) among the three peri-urban districts and one (Sangthong) among the two rural districts (simple random samplings). Differentiation of the districts into urban, peri-urban and rural was based on previous publications [15, 16].

In a previous study in Zimbabwe, the prevalence of citizens unaware of the potential harm induced by OTC drugs was 65 % [7]. Based on this prevalence, we aimed to include 87 to 350 villagers, allowing a precision between 5 and 10 %. This interval was conditioned by the lack of availability of skilled interviewers with medical or pharmaceutical degree. We calculated that three interviewers could interview 10 villagers per village and per day. With an estimated rate of refusal of 20 %, 12 villagers were thus included per village. Three villages per district (this number was chosen for convenience) were randomly selected by simple sampling among the villages with at least one health facility (health centre, hospital, private clinic or private pharmacy), representing 8.2 % (*n* = 12) of all the eligible villages (*n* = 146) in the four surveyed districts.

In the villages where a list of the households was available (*n* = 8), 12 households were selected by systematic random sampling. Systematic sampling involves a random start and then proceeds with the selection of every *k*th element from then onwards [17]. For each village, *k* was calculated as the total number of households divided by 12. In the other villages (*n* = 4) a list of ‘clusters of households’ was provided by the head of the village. From this list, 12 clusters were selected by systematic sampling and in each cluster one household was then chosen at the time of the survey by the research team. The head of the household or his/her wife/husband was interviewed if available at the time of the visit. When s/he was not available, another member of the household was interviewed (≥16 years old).

### Questionnaire and data collection

The questionnaire was developed for the purpose of the study. It was structured into four sections related to: sociodemography, medicinal products use, awareness of medicinal product risks (including traditional medicines, modern medicines and poor quality medicines) and experience of ADR (Table 1).

**Table 1:** Questionnaire for survey of population awareness of risks related to medicinal products use

	Answers			
A. Sociodemographic characteristics				
1. Gender	o Male		o Female	
2. Age	____			
3. Status in the household	o Head of household		o Spouse or husband of head of household	
	o Other, specify ________			
4. Occupation	o Housekeeper		o Farmer	o Other job
	o Retired/disabled		o Student	
5. Monthly household income (Lao kip)	o ≤ 1 000 000		o >1 000 000	
6. Religion	o Buddhism	o Animism	o Other, specify _______	
7. Level of education	o Secondary school or higher		o Primary school or no education	
8. Can you read?	o Yes		o No	
9. Do you have easy access to internet?	o Yes		o No	
B. Medicinal product use				
1. Have you ever consumed modern medicine(s)?	o Yes		o No	
Was the last time during the last 12 months?	o Yes		o No	
If yes, name(s) of the medicine(s) and its (their) indication(s)			________ ________	
2. Have you ever consumed traditional medicine(s)?	o Yes		o No	
Was the last time during the last 12 months?	o Yes		o No	
If yes, name(s) of the medicine(s) and its (their) indication(s)			________ ________	
C. Awareness of medicinal product risks				
1. Do you think that modern medicines can be harmful in case of normal use and normal doses?	o Yes		o No	o Do not know
Comment(s)_____________________________________________				
2. Do you think that modern medicines can be harmful in case of overdosage?	o Yes		o No	o Do not know
3. Do you think that traditional medicines can be harmful in case of normal use and normal doses?	o Yes		o No	o Do not know
4. Do you think that traditional medicines can be harmful in case of overdosage?	o Yes		o No	o Do not know
5. Have you ever heard what an adverse drug reaction is?	o Yes		o No	
Comment(s)_____________________________________________				
6. Have you ever heard of poor quality medicines?	o Yes		o No	
If yes, do you think that poor quality medicines can be harmful?	o Yes		o No	o Do not know
Comment(s)_____________________________________________				
7. Where have you heard of poor quality medicines?	o Healthcare providers		o Magazine, journal	
	o TV		o Relative, friend	o Other, specify ________
D. Experience of adverse drug reaction				
1. Have you (or one of your relative) ever taken a medicine (modern medicine, traditional medicine) that made you feel worse?	o Yes		o No	
If yes, please describe the reaction and the name(s) of the medicine(s)_________________________				
A Pharmacovigilance system is a ‘drug monitoring’ system. This system works through spontaneous reporting of drug-related problems by health care professionals and patients.				
2. Would you agree to notify a drug-related problem to the national pharmacovigilance center?	o Yes		o No	

Most of the questions were closed-ended questions (yes/no or yes/no/do not know) and a few were open-ended. The open-ended questions were read without mentioning any predetermined answers, and the information collected was coded based on identified thematic areas.

Two questions were used to define the awareness of modern medicine risks. The first one was *‘Have you ever heard what an ADR is?’.* In addition, in order not to underestimate the awareness of the population, a second question was added *‘Do you think that modern medicines can be harmful in case of normal use and normal doses?’*. This latter was adapted from another study [7]*.* In order to verify their understandings of these two questions, the positive respondents were invited to give comments.

A negative answer to the first question (ADR) and a negative or a ‘do not know’ answer to the second one were the criteria to consider a villager as unaware of medicine risks.

The questionnaire was developed in English and translated into Lao by a Laotian pharmacist with strong background of English practice. The technical words were checked by two Laotian pharmacists specialized in public health. The questionnaire was then back-translated into English by a Lao medical doctor with a Tropical and International Health degree to make sure the first translation was accurate. After translation and back-translation, no discrepancies for the questions used for the measures of the outcome were highlighted. We piloted the questionnaire with four villagers (heads of household or their spouse/husband) living in Vientiane to assess understanding and ease of completion. The respondents to the pilot survey were asked to tell if all the words were understood and they were also asked to explain in details the choices of their answers, with a particular attention to the questions of the Section C and D.

The questionnaires were administered by face-to-face interviews by three trained physicians using local language (i.e. Lao), either in the office of the head of the village (in two villages), at the temple of the village (in one village), or within the households (in nine villages).

### Data analysis

Characteristics of those unaware versus aware of medicine risks were compared using χ^2^ tests when all expected counts were above five, or otherwise with Fisher’s exact tests. T-tests were used to compare quantitative characteristics if the equality of the variances was confirmed by F-tests. If variances were not equal, Wilcoxon tests were used.

To identify factors associated to the unawareness of medicine risks, logistic regression was performed. The following socio-economic characteristics were tested: gender, age, educational level, district, monthly household income, status within the household, occupation, ability to read and access to internet. The variables with a *p*-value <0.20 after univariate analysis were entered into a multivariate logistic regression model.

The goodness of fit of the final model was assessed using the Hosmer and Lemeshow test. The results are presented as odds-ratio (OR) or adjusted odds ratios (aOR) and their 95 % confidence intervals (CI). Data analysis was carried out using SAS® 9.3 software (SAS Inst., Cary, North Carolina, USA). The level of significance was set at 0.05 (two-sided).

### Ethical approval

Written informed consent was obtained from all participants. Ethical clearance for the study was granted by the Ethics Committee of the University of Health Sciences, Ministry of Health of Laos.

## Results

### Respondent characteristics

A total of 144 Lao citizens were solicited and all consented. An equal number of respondents in each selected district (*n* = 36) were included. More women (*n* = 108) than men (*n* = 36) were included and the mean age was 45.1 (SD 12.5) years old. One-third of all respondents (*n* = 47) were the heads of the household, with a majority of men (*n* = 32). Half (*n* = 74) were their spouses.

A total of 89 [61.8 % (95 % CI = 53.8–69.8)] respondents were unaware of modern medicine risks (Table 2). Respondents being unaware tended to have a lower level of education than those being aware (55.1 vs 34.6 %, *p* = 0.03). There were differences according to the district (*p* < 0.001). Respondents being unaware tended to live more in the rural district of Sangthong (33.7 vs 10.9 %, *p* < 0.01 respectively) and less in the urban district of Xaysetha than those being aware (15.7 vs 40.0 %, *p* < 0.01 respectively). The proportions of respondents living in Sikhottabong (urban) or in Naxaithong (peri-urban) were not statistically different between the respondents being aware and unaware.

**Table 2:** Socio-demographic characteristics of the respondents (*N* = 144) according to the awareness of medicine risks

	Aware of medicine risks n (%)	Unaware of medicine risks n (%)	*p*-value*
**Total**	55 (38.2)	89 (61.8)	
**Age (years)**			0.07
Mean ± SD	42.7 ± 10.6	46.5 ± 13.4	
**Gender**			0.24
Male	17 (30.9)	19 (21.4)	
Female	38 (69.1)	70 (78.6)	
**Status in the household**			0.76
Head of household	16 (29.1)	31 (34.8)	
Husband/spouse of the head of household	29 (52.7)	45 (50.6)	
Other	10 (18.2)	13 (14.6)	
**Occupation**			0.10
In employment (except farmers)	35 (63.6)	42 (47.2)	
Housekeeper	13 (23.6)	24 (27.0)	
Farmer	7 (12.7)	23 (25.8)	
**Religion**			0.52
Buddhism	55 (100.0)	87 (97.8)	
Animism	0	2 (2.3)	
**Monthly household income (Lao Kip)**			0.13
>1,000,000	7 (13.0)	21 (24.1)	
≤1,000,000	47 (87.0)	66 (75.9)	
**Level of education**			0.03
Secondary or higher	36 (65.5)	40 (44.9)	
No education or primary school	19 (34.6)	49 (55.1)	
**Can read**			0.74
Yes	52 (94.6)	82 (92.1)	
No	3 (5.5)	7 (7.9)	
**Access to internet**			0.08
Yes	8 (14.6)	5 (5.6)	
No	47 (85.5)	84 (94.4)	
**District**			0.001
Xaysetha	22 (40.0)	14 (15.7)	
Sikhottabong	15 (27.3)	21 (23.6)	
Naxaithong	12 (21.8)	24 (27.0)	
Sangthong	6 (10.9)	30 (33.7)	

*χ^2^, Fisher’s tests (qualitative characteristics) or t-test (quantitative characteristics)

### Medicinal product use

All the respondents claimed to have used a modern medicine and 87 (60.4 %) a traditional medicine during their lifetime. One-hundred and two (70.8 %) respondents had used at least one medicine (modern or traditional) within the last 12 months. Fifty-one (50.0 %) of them could not give the names of the last modern medicines used within the previous 12 months (Table 3). The main indications of these unidentified modern medicines were pain (*n* = 17), fever (*n* = 6), diabetes (*n* = 4), allergy (*n* = 3), cough (*n* = 3), contraception (*n* = 2), hypertension (*n* = 2) and hypercholesterolemia (*n* = 2). Among the modern medicines that the respondents were able to name, analgesics and vitamins were the most frequently used. The analgesics were paracetamol or paracetamol in combination with antihistamine and sympathomimetic agents (*n* = 14), non-steroidal anti-inflammatory drugs (*n* = 3), and metamizole (*n* = 1).

**Table 3:** Last medicines used within the previous 12 months by the 102 respondents

Medicines	n (%)
Unknown/unidentified	51 (50.0)
Analgesics	18 (17.6)
Vitamins/minerals	14 (13.7)
Traditional medicines	13 (12.7)
Anti-infectious	10 (9.8)
Medicines of gastro-intestinal disorders	8 (7.8)
Cardiovascular medicines	5 (4.9)
Antidiabetics	5 (4.9)
Other	2 (2.0)

None of the 15 respondents could give a name for the traditional medicines. However, most could give a description of the part of the plant used [(e.g. ‘*leaves of a tree’* (*n* = 3) or ‘*roots of a tree’* (*n* = 6)] and its indication [mainly pain (*n* = 5), diabetes (*n* = 2) and gastritis (*n* = 2)].

### Factors potentially associated with being unaware vs being aware of medicine risks

Occupation was not included in the multivariate regression model because of a strong collinearity with the district. Indeed, there were significantly more farmers in the rural and peri-urban districts as compared to urban ones (38.6 % and 36.1 % vs 4.2 %, respectively, *p* < 0.01).

In the multivariate logistic regression model, the district was the only significant variable potentially associated with an increased risk of being unaware of risks related to medicine use. Compared to the respondents of the urban district of Xaysetha, the respondents in the peri-urban (Naxaithong) and even more in the rural district (Sangthong) were more likely to be unaware of medicine risks [ aOR =3.3, (95 % CI =1.1–9.4) and aOR =7.5 (95 % CI = 2.3–24.2), respectively] (Table 4). The respondents in the rural district were also more likely to be unaware of medicine risks compared to those living in the urban district of Sikhottabong [aOR = 4.5 (95 % CI = 1.3–15.1)] but no statistical differences were observed between the respondents in Sikhottabong and those in the peri-urban district of Naxaithong [aOR = 1.9 (95 % CI = 0.7–5.7)].

**Table 4:** Factors influencing the awareness of the population regarding medicines risks: ‘unaware’ vs ‘aware’. Results of univariate and multivariate analysis

	Univariate analysis		Multivariate analysis	
Variables	OR (95 % CI)	*p*-value	Adjusted OR (95 % CI)	*p*-value
**Age group (years)**		0.10		0.11
≤40	1		1	
[40–50]	0.9 (0.4–2.0)		0.8 (0.3–2.0)	
>50	2.3 (0.9–5.5)		2.3 (0.8–6.7)	
**Gender**		0.21		
Male	1			
Female	1.6 (0.8–3.5)			
**Status in the household**		0.72		
Head of household	1			
Husband/spouse of the head of household	0.8 (0.4–1.7)			
Other	0.7 (0.2–1.9)			
**Occupation**		0.10		
In employment (except farmer)	1			
Housekeeper	1.5 (0.7–3.5)			
Farmer	2.7 (1.1–7.1)			
**Religion**		0.99		
Buddhism	1			
Animism	N/A			
**Monthly household income (Lao Kip)**		0.11		0.06
>1,000,000	1		1	
≤1,000,000	2.1 (0.8–5.4)		2.8 (1.0–8.1)	
**Level of education**		0.02		0.56
Secondary or higher	1		1	
Primary school or no education	2.3 (1.2–4.7)		1.3 (0.6–2.9)	
**Ability to read**		0.58		
Yes	1			
No	1.5 (0.4–6.0)			
**Access to internet**		0.08		0.22
Yes	1		1	
No	2.9 (0.9–9.2)		2.3 (0.6–8.5)	
**District**		<0.01		<0.01
Xaysetha	1		1	
Sikhottabong	2.2 (0.9–5.6)		1.7 (0.6–4.8)	
Naxaithong	3.1 (1.2–8.2)		3.3 (1.1–9.4)	
Sangthong	7.9 (2.6–23.7)		7.5 (2.3–24.2)	

*OR*, odds-ratio; *CI*, confidence interval

The occupation factor was not included in the multivariate model because of its strong collinearity with the district localization

### Awareness of medicinal product safety

Among the 55 respondents who were qualified as aware of modern medicine risks, 53 (96.4 %) had heard what an ADR is. These respondents gave examples of symptoms of adverse reactions. The most commonly quoted were allergic and gastro-intestinal symptoms. Sometimes the respondents associated the culprit medicine to the symptom (e.g.: *‘ampicillin can cause allergy’*). Some respondents also tried to define what an ADR is (ex: ‘*it is when new symptoms appear when you take a medicine*’). Of these 53 respondents, a majority (*n* = 45, 84.9 %) said that modern medicines are potentially harmful in overdosage, but only five (9.4 %) believed that they can be harmful at recommended doses.

Regarding traditional medicines, 83 (57.7 %) participants believed that they are not potentially harmful with normal doses and with normal use, and about half of them (n = 39), representing 27.1 % of all the participants, thought that they are safe in overdosage. In addition, 46 (31.9 %) and 54 (37.5 %) respondents had no opinion on the potential noxiousness of traditional medicines in case of normal use and in overdosage, respectively.

### Awareness of the existence of poor quality medicines

One half (*n* = 68, 47.2 %) of the respondents had heard of poor quality medicines, mainly through media (TV, radio, papers; *n* = 41, 60.3 %) or through relatives/friends (*n* = 16, 23.5 %). Those living in peri-urban/rural districts were significantly less aware of their existence in comparison to those in urban districts (38.9 % vs 55.6 %, respectively, *p* = 0.02). In response to the question *‘Do you think that poor quality medicines can be harmful?’*, most of the respondents (*n* = 59, 86.8 %) answered yes and 8 (11.8 %) had no opinion. Respondents were invited to comment on this latter question. Among the respondents who gave a comment (*n* = 40), 18 (45 %) expressed concerns regarding the risk of inefficacy of the poor quality medicines, 11 (27.5 %) about the potential occurrence of ADRs (e.g. ‘*harmful for the liver’*, ‘*harmful for the kidney*’) and 6 (15 %) expressed both concerns of inefficacy and potential occurrence of ADRs.

### Experience of adverse reaction to modern or traditional medicines

Fifty-two (36.1 %) respondents claimed to have encountered a medicine that *‘made them or their relative feel worse’*, most of them being aware of medicine risks (60 % versus 21 % being unaware, *p* < 0.001). All described the reaction observed, but only half (*n* = 28) could give the name or the pharmacological class of the medicine involved, an antibiotic being involved in 18 cases (64.3 %). Symptoms of allergy such as ‘rash’, ‘shock’, ‘dyspnea’ or ‘oedema’ were described by 35 (67.3 %) respondents among whom five were serious adverse reactions (e.g. angioneurotic oedema). Forty percent of the cases of allergy involved antibiotics (*n* = 14), one case involved the *‘leaves of a tree’* and 15 (42.9 %) respondents did not know the name of the medicine(s) involved. Gastro-intestinal symptoms (9 cases, 17.3 %) and general symptoms such as ‘*fatigue*’ or ‘*dizziness*’ (15 cases, 28.9 %) were also noted. A total of 140 (97.2 %) respondents would agree to notify an ADR to the concerned authority in the future.

## Discussion

In this population-based study, the majority of the respondents were unaware of modern medicine risks. Most of the respondents qualified as aware had an inadequate awareness since most thought that modern medicines can be harmful only in overdosage. As for modern medicines, the awareness of traditional medicine risks was limited. Half of the participants were not aware of the existence of poor quality medicines in the market.

The median age (47 years old) of our population is higher than that of the population at national level since 75 % of people were aged between 15 and 45 years old in 2005 [18]. More women than men were interviewed mainly because interviews were performed during working hours. Nevertheless, even if our population does not strictly represent the general population of lao adults, 85 % of the respondents were the heads of household or their husband/spouse, who play an important role in healthcare choices for the whole family in L/MICs [19–21]. No citizen refused to participate in the study. Government staffs have a strong influence on citizens in the Lao PDR. The 100 % response rate can thus result from the first contact of the citizens that was made by the head of the village or his mandated assistant. Whereas these latter were present during some interviews, they agreed to stay apart. However, when the interviews were performed in the households it was difficult to maintain strict confidentiality, as other family members or neighbours sometimes came to listen.

In our survey, only one-fourth of the population is rural. As 71 % of the population of the Lao PDR lived in rural areas in 2007/2008 [22], we can reasonably assume that the awareness of the general population of Lao PDR regarding the issue of medicinal product risks is lower than that observed in our study. This is highlighted by the significant differences of awareness observed between the districts, respondents in the rural district having a higher risk of being unaware than those in urban districts. Further data [18, 23] have shown that the distribution of health facilities was different according to the districts. Private clinics – run by medical doctors with university degree –, and class I community pharmacies – run by pharmacists with university degree –, are more prevalent in the urban districts of Xaysetha (84.1 and 40.0/100,000 inhabitants, respectively) and Sikhottabong (44.0 and 13.0/ 100,000 inhabitants respectively) than in the peri-urban district of Naxaithong (30.8 and 1.7 /100,000 inhabitants, respectively) and in the rural district of Sangthong (24.8 and 4.1/100,000 inhabitants, respectively). Therefore, we suggest that whereas people in rural areas have access to health facilities, urban residents have a better opportunity to receive information from healthcare providers of higher level of training. Whereas respondents in the urban district of Sikhottabong have a significant higher awareness than those in the rural district of Sangthong, there were no differences with those in the peri-urban district of Naxaithong. This could be explained by the distribution of health facilities in Sikhottabong that is in between that of the peri-urban district of Naxaithong and of the urban district of Xaysetha. In addition, as suggested by Syhakhang et al. [16], people in urban areas may have better opportunity to receive information on medicines from the regulatory authority. More investigations are needed to assess other potential factors that might explain the differences between the districts.

In the absence of validated questionnaire to assess the awareness of the population on medicine risks when they are used with normal doses, our questionnaire was developed for the purpose of this study. While its reliability and validity have not been tested, the questionnaire was submitted to four experts in public health, epidemiology and pharmacology for an opinion, before being translated into Lao. The term ADR is defined by the WHO as *“a response to a drug which is noxious and unintended and which occurs at doses normally used in humans”*[24]*.* It is used for communication on drug safety in validated sources of information on medicines (i.e. summary of products characteristics and leaflets). However, when this term has never been heard by a villager, it does not necessarily mean that he is not aware of medicine risks. We thus combined this question to a second one (*‘Do you think that modern medicines can be harmful in case of normal use and normal doses?’*) to define our outcome of interest.

We observed that even when they were qualified as aware of medicine risks, the large majority of our respondents think that modern medicines are potentially harmful only in overdosage. In addition, the majority of the respondents in our study considers traditional medicines as safe or has no opinion, even in overdosage. Therefore, strategies to raise awareness of the population on drug safety, without scaring patients from using medicinal products are needed, with special attention to rural areas. This is particularly important in a country where most medicines, even the most harmful, are readily available OTC. Mass media campaigns have previously shown promising results in health promotion in L/MICs [25, 26]. Adequate key messages on drug safety, for example targeting medicinal products commonly involved in ADRs could be relevant. For this purpose, drug safety data in the Lao PDR are needed.

Half of our respondents had never heard of poor quality medicines. Although efforts made by the government since the implementation of the National Drug Policy [8] have allowed reduction in the proportion of poor quality medicines in the market [27], the threat is still real [13]. Most of the people being aware of poor quality medicines had been informed through media in our study. Initiatives to raise awareness of the population such as the ‘Mekong Cartoon Contest’ in 2011 (“Beware of counterfeiting, danger is calling!”) with online diffusion of the cartoons [28], need to be continued.

Half of our participants could not name the last medicines used within the last year. This can be due to recall bias, illiteracy concerns or non-adherence to prescribed medications (with regard to chronic diseases medications). This could also result from a lack of identification of medicines, as previously observed in two studies in which half of the medicines sold in private or public pharmacies had no label [9, 29]. As accepted important sources of information on drug use and safety [30], the provision of labels and package inserts should be endorsed. It should be mentioned that a new article (2012) of the Laotian law on drug and medical products states that medicine users should receive clear and complete information from medicines suppliers. However, package inserts are often written in medical jargon and too many information is generally provided. They are thus difficult to understand [31]. When printed in foreign languages, they can be incomprehensible for patients, especially in rural areas [7]. Distributed by pharmacists and other health professionals, well-adapted model sets of minimum information developed in a way that is relevant to each population – e.g. using regional languages and illustrations [32] – have previously shown good effectiveness [33, 34]. The best effects on patients knowledge are evident when both written and oral information on medicines are provided [35]. In a previous study by Stenson et al. [9], oral information were not transmitted for more than half of medicines sold in laotian pharmacies of low level (class III). Engaging all healthcare professionals in providing adequate oral information is essential – especially in the context of high illiteracy – , not only to improve patients’ understanding of their medication, but also to build trustworthy health system [36–38]. Enhancing the training of pharmacist and medical students on drug safety communication seems necessary in the Lao PDR.

In line with the efforts to improve the use of medicines, the Lao PDR has recently (2013) become an associate member of the WHO Programme for International Drug Monitoring [39]. Drug safety monitoring requires the involvement of healthcare professionals. In addition, central, provincial or district level drug information centers for patients who seek information on medicines could help transforming medicine users from passive recipients to active partners [40–42]. In remote areas, community health workers or community leaders have proved highly effectiveness in promoting health [43, 44] and could constitute mediators between the population and a drug information center. At last, mobile technologies and web-based systems have recently proved good performance in health care processes in Asia [45, 46].

## Conclusion

We have demonstrated concerns about the awareness of the risks of medicinal products among the population of Vientiane Capital. Differences according to the place of residence are apparent and could be partly explained by a lower level of training of healthcare providers in contact with the population in the rural districts in particular. The most important recommendation would be to invest more in education and information about these risks for the population. This could make a valuable contribution towards the improvement of the appropriate and correct use of medicines, and thus towards drug safety, in Lao PDR and in L/MICs in general.

## Abbreviations

L/MICs: Low- and middle-income countries
ADRs: Adverse drug reactions
PDR: People’s Democratic Republic
WHO: World Health Organization
OR: Odds ratio
OTC: Over-the-counter
